# Treatment of heterotopic cervical pregnancy by ultrasound-guided hysteroscopy: A case report and literature review

**DOI:** 10.1097/MD.0000000000032177

**Published:** 2022-12-02

**Authors:** Shuman Sheng, Haomeng Zhang, Zhengwu Pan, Tao Li, Xin Wang, Min Shi, Fei Wang

**Affiliations:** a Department of Obstetrics and Gynecology, Shandong Provincial Hospital Affiliated to Shandong First Medical University, Jinan, Shandong, People’s Republic of China; b Department of Obstetrics and Gynecology, Shandong Provincial Hospital, Shandong University, Jinan, Shandong, People’s Republic of China; c Department of Ultrasound Medicine, Shandong Provincial Hospital Affiliated to Shandong First Medical University, Jinan, Shandong, People’s Republic of China

**Keywords:** case report, cervical pregnancy, ectopic pregnancy, heterotopic pregnancy, hysteroscopy, ultrasound

## Abstract

Heterotopic cervical pregnancy is a rare event of ectopic pregnancy with an incidence rate of < 1%. Herein, we report a rare case of successful treatment of heterotopic pregnancy following an *in vitro* fertilization-embryo transfer using ultrasound-guided hysteroscopy. In order to choose the best treatment option, we reviewed the clinical treatments and discussion of heterotopic cervical pregnancy over the last 15 years.

**Methods:**

The heterotopic pregnancy was terminated using ultrasound-guided hysteroscopy; however, the intrauterine pregnancy was maintained. We searched for the keywords “cervical pregnancy combined with intrauterine pregnancy,” “compound pregnancy,” “assisted reproductive technology,” “cervical pregnancy,” and “ectopic pregnancy” on PubMed to include articles published in the last 15 years.

**Results:**

The patient underwent an emergency cervical cerclage at 22 weeks’ gestation for cervical insufficiency and delivered a healthy newborn at 38 weeks’ gestation by transvaginal compliance. Twenty-one relevant case reports were selected. After analysis and discussion, we found that assisted reproductive technology is more likely to lead to heterotopic pregnancy than unassisted reproduction. Most women requesting the preservation of intrauterine embryos opted for surgical termination of cervical pregnancy and achieved the ideal outcomes.

**Conclusion:**

More attention should be paid to the diagnosis and treatment of heterotopic pregnancies to obtain the most optimal pregnancy outcome and long-term prognosis. Hysteroscopic surgery is a completely feasible cervical pregnancy treatment option with less postoperative impact on the mother and the intrauterine fetus.

## 1. Introduction

Ectopic pregnancies have become more common as assisted reproductive technology (ART) has advanced. According to one study, the incidence of ectopic pregnancy in women who have undergone ART is between 2.1% and 8.6%, while the incidence in women who have spontaneous pregnancy is 2%.^[1]^ Heterotopic pregnancy is a special type of ectopic pregnancy, which refers to the implantation of embryos in more than 2 different sites; the incidence thereof accounts for 1 to 3%.^[2]^ The most common type of heterotopic pregnancy is intrauterine pregnancy combined with tubal pregnancy, while intrauterine pregnancy combined with cervical pregnancy is rare. The diagnosis of heterotopic pregnancy is confirmed using transvaginal ultrasound and pelvic nuclear magnetic resonance examination.^[3–7]^ There are no clear management guidelines for intrauterine pregnancy combined with cervical pregnancy, treatment is based on the woman’s willingness to continue the pregnancy and the clinical experience of the attending physician.

We report a case of heterotopic cervical pregnancy and discuss the treatment and outcomes of heterotopic pregnancy after searching for related case reports published over the past 15 years. Before the completion of this manuscript, the patient had successfully delivered a healthy newborn. We obtained informed consent forms and the woman gave her consent for her images and other clinical information to be reported.

## 2. Case report

**Figure 1.**
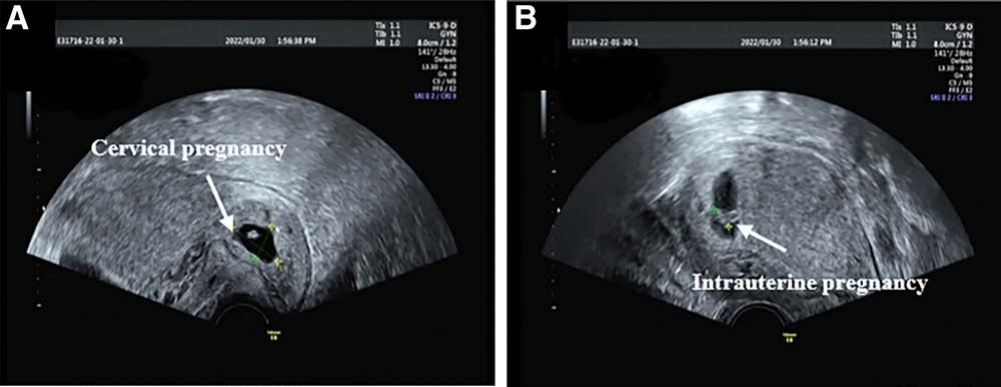
Transvaginal ultrasonography on day 33 of embryo transfer. (A) The pregnancy sac in the cervical canal, yolk sac, and fetal heartbeat are visible; fetal bud length is approximately 0.6 cm. (B) The intrauterine pregnancy sac, yolk sac, and fetal heartbeat are visible; the fetal bud is approximately 0.9 cm.

A 31-year-old gravida 2 para 0 (G2P0) woman had undergone left salpingectomy for tubal pregnancy and lost her left fallopian tube. Additionally, she had hysteroscopic surgery for multiple endometrial polyps. She received *in vitro* fertilization embryo transfer and 2 fresh embryos were transplanted in December 2021. After the embryo transfer, the woman was treated with dydrogesterone and progesterone. She developed light, painless vaginal bleeding due to abdominal exertion 27 days after the embryo transfer. However, ultrasonography did not show any abnormalities. Ultrasound imaging performed 33 days after the embryo transfer revealed that she had cervical and intrauterine pregnancies (Fig. 1). Her hematological indicators were as follows: serum beta-human chorionic gonadotropin (β-HCG) level, 119,885 mIU/L; hemoglobin level, 144 g/L; and white blood cell count, 11.24 × 10^9^ cells/L.

**Figure 2.**
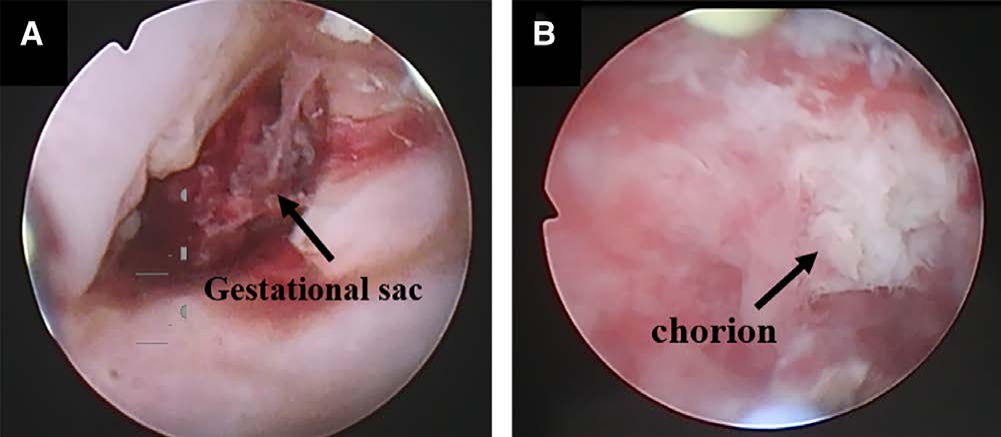
Preoperative and postoperative hysteroscopic images. (A) Hysteroscopic image of cervical pregnancy. A blood clot of approximately 1 cm in diameter is seen at the cervix. (B) Hysteroscopic image of the cervical canal after the surgery.

The woman and her spouse requested that the intrauterine pregnancy should be preserved. We decided to perform ultrasound-guided hysteroscopic surgery. On the second day of admission, the woman underwent hysteroscopic surgery (Olympus OTV-S190) under ultrasound monitoring (NAVIS) and intravenous general anesthesia. Transabdominal ultrasonography was performed before the surgery and a pregnancy sac was detected in the uterine cavity and cervical canal. In both pregnancies, we observed a fetal bud and heartbeats. All the subsequent surgical steps were performed under ultrasound monitoring. After exposing the cervix, we detected a blood clot of approximately 1 cm in diameter at the internal cervix os. The hysteroscopic pressure was set to 100 mm Hg and the flow rate to 100 mL/min to avoid excessive expansion of fluid into the uterine cavity and adverse effects on the uterine pregnancy. We carefully removed the blood clot and performed a hysteroscopy to explore the cervix. We found a dark red gestational sac in the cervix with poor tension and an unclear boundary with mucosa of the cervical canal (Fig. 2a). A circular electrode was used to remove the villi in the cervical lumen. During this process, ultrasound was used to monitor the fluid accumulating in the uterine cavity through the internal cervical os. When the ultrasonography showed that the fluid occupied approximately one-third of the volume of the uterine cavity, the hysteroscopy was stopped and the internal cervix os was slightly dilated with oval forceps and the accumulated fluid was seen flowing out of the cervix.

When the ultrasound monitoring showed that the uterine distention has improved, cervical lavage with the hysteroscope was initiated until the cervical pregnancy was completely removed. A Foley catheter was placed into the cervical lumen, and 2 pieces of iodine gauze were inserted into the vaginal fornix. There was slight intraoperative bleeding, and no blood transfusion was required. Ultrasonography and hysteroscopy showed no significant residue in the cervix (Fig. 2b). The fetal heart rate was detected at 160 bpm using ultrasound during the surgery, which ended without adverse events.

**Figure 3.**
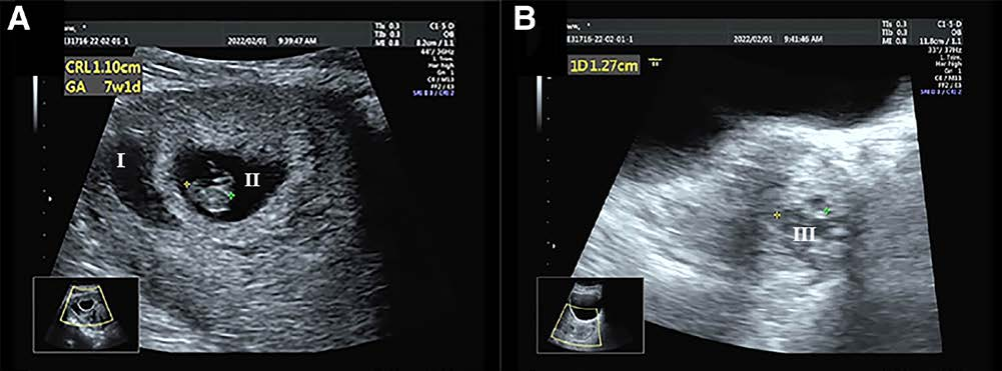
Transabdominal ultrasonography on postoperative day 1. (A) The intrauterine pregnancy sac, fetal bud, and fetal heartbeat can be seen (I); the length of the fetal bud is approximately 1.1 cm. The dark liquid area around the gestational sac is approximately 3.2 × 3.1 × 2.2 cm, with poor internal sound transmission and a ground-glass appearance (II). (B) A small amount of dark liquid area of 1.4 × 1.3 × 1.0 cm can be seen in the upper part of the cervix (III), and a separated echo can be seen.

**Figure 4.**
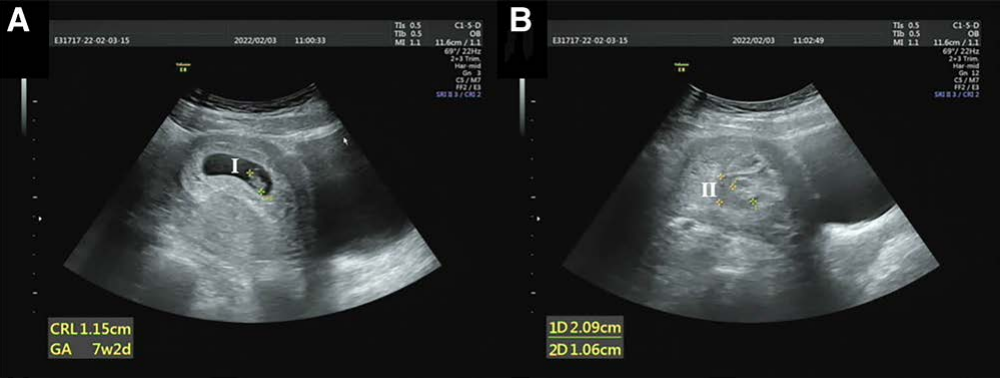
Transabdominal ultrasonography on postoperative day 7. (A) The intrauterine pregnancy sac, yolk sac, fetal bud, and fetal heartbeat are visible (I); the length of the fetal bud is approximately 1.8 cm. (B) A dark fluid area of approximately 2.6 × 1.2 × 2.2 cm is detected in the lower uterine cavity below the pregnancy sac with poor internal sound penetration (II). No abnormal echo is found in the cervix.

On postoperative day 1, the Foley catheter and 2 iodophor sponges were removed from the cervix, and we detected no abnormal signs. Ultrasound reexaminations on postoperative days 1, 3, and 7 are shown in Figures 3–4. The woman was discharged on postoperative day 7.

A postoperative routine pathological examination of the surgical specimen revealed uterine pregnancy and placental villus tissue. At 22 weeks of gestation, the woman was diagnosed with cervical incompetence and she underwent cervical cerclage; at 38 weeks of gestation, she delivered a healthy newborn by cesarean section because of cephalopelvic disproportion. The patient appreciated our previous work.

## 3. Discussion

Ultrasound reports of cervical pregnancy were first reported by *Raski* in 1978.^[8]^ The incidence of cervical pregnancy is low (<1%) compared to the incidence of ectopic pregnancy at other sites^[2]^; however, the risk is relatively high. If left untreated, there is a risk of hemorrhage, hysterectomy, or death. These risks are further increased when cervical pregnancy is combined with intrauterine pregnancy and we have to consider the possibility of survival of the intrauterine pregnancy during treatment. *Matorras, et al* and *Moragianni, et al* identified the main risk factors for cervical pregnancy as previous pregnancy, miscarriage, curettage, smoking, and ART.^[9,10]^ Women usually present with vaginal bleeding, with or without lower abdominal pelvic pain, which is usually diagnosed by transvaginal ultrasound.^[10]^ Approximately 70% of heterotopic pregnancies are reported to be diagnosed between 5 and 8 weeks of gestation, 20% between 9 and 10 weeks, and the remaining 10% after 11 weeks of gestation.^[11]^ For the management of cervical pregnancy, *Fan, et al* suggest that treatment selection should be based on gestational age, initial serum β-human chorionic gonadotropin, fetal heart, vaginal bleeding, and the woman’s willingness to preserve fertility.^[4]^ Treatment options are usually surgical and conservative. Surgical treatment includes aspiration, forceps, curettage, hysteroscopy, and uterine artery embolization. Conservative treatment includes local or systemic methotrexate (MTX), potassium chloride (KCl), high concentration sodium chloride (NaCl), or glucose injections. In the case of heterotopic pregnancy, if the woman requests to preserve the intrauterine pregnancy, in addition to the impact of this treatment on maternal health and future fertility, we must consider whether this treatment will cause adverse effects on intrauterine embryos (such as adverse effects from MTX and uterine artery embolization), which are not suitable for life intrauterine embryos. The Canadian Society of Obstetricians and Gynecologists (SOGC) 2021 Clinical Practice Guidelines on ectopic pregnancy recommend that MTX should not be administered for conditionally reserved intrauterine pregnancies and surgical removal of ectopic pregnancies in women with complex pregnancies.

To evaluate the treatment of cervical pregnancy combined with intrauterine pregnancy, we reviewed 21 relevant case reports between 2007 and 2022 (Table 1). We found that, according to the method of the conception of pregnancy, 14.3% (3/21) of women conceived naturally; 85.7% (18/21) of women received assisted reproductive treatment. In terms of pregnancy outcome, 71.4% (15/21) of women achieved a good outcome, with cervical pregnancy removed successfully, a healthy new-born, and fertility preservation. A total of 4.8% (1/21) of women underwent hysterectomy because of complications after successful birth; 19% (4/21) of women chose to terminate the intrauterine and cervical pregnancies. In terms of delivery mode, 18.75% (3/16) of women had natural vaginal birth; 81.25% (13/16) of women chose cesarean birth, among which 7 women had indications (such as breech position, misalignment of the cephalic pelvis, placental prepositioning) for cesarean birth. In terms of gestation weeks, 75% (12/16) of women gave birth after full term (37 w); 25% (4/16) of women had premature birth due to complications (such as premature rupture of membranes massive hemorrhage, and abruptio placentae). In terms of choice of treatment method, 61.9% (13/21) of women underwent surgical methods (such as aspiration, clamp, hysteroscopy, uterine artery embolization) to remove cervical pregnancy; cervical pregnancy was removed by medication (such as MTX, high concentration sodium chloride, and high concentration glucose) in 14.3% (3/21) of women and by combined surgery and conservative treatment in 23.8% (5/21) of women. According to the above data, it can be determined that the incidence of heterotopic pregnancy in women who have received assisted reproductive treatment is significantly higher than in women who have spontaneous pregnancies. At present, the method of diagnosis and treatment of heterotopic pregnancy is well established, and it can be detected early with rapid intervention. In terms of treatment, most women who requested the preservation of intrauterine embryos opted for surgical termination of cervical pregnancy and achieved the ideal outcomes: termination of cervical pregnancy, successful preservation of intrauterine pregnancy, and future fertility. Although the rate of cesarean section appeared to be elevated, there was no significant correlation with the management of cervical pregnancy.

**Table 1:** Overview of published cervical heterotopic cases (presented in reverse chronological order of publication).

Reference	GA at diagnosis	Method of conception	Treatment	Outcome
Current case	6 wk + 6 d	IVF-ET	Hysteroscopic resection (ultrasound-guided); Foley catheter	Follow-up
Fan et al^[4]^	42 d after ET	ICSI-ET	Aspiration (ultrasound-guided); tranexamic acid gauze	Delivery at 39 wk (healthy NB, c/s)
Fan et al^[4]^	36 d after ET	IVF-ET	Aspiration (ultrasound-guided); tranexamic acid gauze	Delivery at 27 wk (neonatology treatment, healthy NB, vaginal)
Jin et al^[12]^	7 wk + 2 d	IVF-ET	Laparoscopic temporary bilateral uterine artery occlusion	Delivery at 39 wk (healthy NB, c/s)
Koutras et al^[13]^	7 wk + 4 d	Spontaneous	Mifepristone and misoprostol; curettage; Foley catheter; ligation of the branches of the cervical arteries	Termination; hemorrhage
Sepulveda Gonzalez et al^[14]^	7 wk + 3 d	Stimulation IUI	Laser ablation (ultrasound-guided)	Delivery at 36 wk (healthy NB, c/s)
Terra et al^[15]^	7 wk *+* 5 d	IVF-ET	KCl injection (ultrasound-guided); curettage	Delivery at 39 wk (healthy NB, c/s)
Bhairavi et al^[16]^	6 wk	IVF-ET	Aspiration (ultrasound-guided)	Delivery at 37 wk (healthy NB, c/s)
Bhairavi et al ^[16]^	6 wk	IVF-ET	Aspiration (ultrasound-guided)	Delivery at 37 wk (healthy NB, c/s)
Saito et al^[17]^	5 wk + 2 d	FET	Extraction with forceps (ultrasound-guided)	Delivery at 36 wk (healthy NB, c/s); total placenta accreta; hysterectomy
Punhani et al^[11]^	8 wk	Stimulation/ICSI-FET	KCl injection; UAE; MTX	Termination
Subedi et al^[18]^	21 d after ET	IVF-ET	UAE; Hysteroscopic	Termination
Pinto et al^[19]^	6 wk + 2 d	Stimulation/ISCI-ET	Extraction with curettage	Delivery at 39 wk (healthy NB, c/s)
Tsakos et al^[20]^	5 wk + 3 d	IVF-ET	Aspiration (ultrasound-guided); Foley catheter; cervical cerclage	Delivery at 38 wk (healthy NB, c/s)
Moragianni et al^[10]^	7 wk 3 d	CC/IUI	Extraction with forceps; Foley; cervical cerclage (48 hrs)	Delivery at 39 wk (healthy NB, c/s)
Faschingbauer et al^[21]^	9 wk	Stimulation	Suction curettage; cervical cerclage	Delivery at 39 wk + 3 d (healthy NB, vaginal)
Sijanovic et al^[22]^	6 and 7 wk	Spontaneous	Local treatment of MTX	Delivery at 39 wk (healthy NB, vaginal)
Shah et al^[23]^	7 wk	IVF-ET/ICSI	Aspiration (ultrasound-guided); bilateral hypogastric artery occlusion balloons	Delivery at 37 wk (healthy NB, c/s)
Kim et al^[24]^	8 wk	Spontaneous	Aspiration (ultrasound-guided); Foley catheter	Delivery at 37 wk (healthy NB, c/s)
Prorocic and Vasiljevic^[25]^	6 wk (2 intrauterine, 1 cervical)	IVF-ET	Aspiration and KCl injection (ultrasound-guided); ligation of descending cervical branches of uterine arteries	Doing well at 12 wk
Suzuki et al ^[26]^	6 wk (2 intrauterine, 1 cervical)	IVF-ET	Aspiration and hyperosmolar glucose injection (ultrasound-guided)	Delivery at 34 wk (healthy NB, twins, c/s); postpartum hemorrhage; large cervical hematoma
Nitke et al^[27]^	7 wk (1 intrauterine, 2 cervical)	IVF-ET	Selective intrauterine artery catheterization; MTX; embolization with Gelfoam	Termination

CC = clomiphene citrate, ET = embryo transfer, FET = frozen-thawed embryo transfer, ICSI = intracytoplasmic sperm injection, IUI = intrauterine insemination, IVF = *in vitro* fertilization, KCl = potassium chloride, NB = new-born; c/s = cesarean section.

Our patient had a previous tubal pregnancy with the removal of one fallopian tube, hysteroscopic surgery, and assisted reproductive treatment, which are all considered risk factors for ectopic pregnancy.^[6]^ The woman developed vaginal bleeding on day 27 after embryo transfer. Considering her willingness to preserve the intrauterine pregnancy, we did not recommend conservative treatment and the use of MTX, because it might have side effects, the most serious of which is fetal teratogenesis. Uterine artery embolization also has the risk of radiation and fetal malformation. Moreover, it has been reported that uterine artery embolization can lead to necrosis of the tissues of the internal cervix os, which leads to severe chorioamnionitis and maternal septicemia.^[28]^ The biggest advantage of hysteroscopic surgery is visibility. Once intraoperative bleeding is found, the origin is readily located, and the bleeding vessel will be ligated. Hysteroscopy has drawbacks, such as cervical dilation, which can lead to intrauterine pregnancy miscarriage, premature birth, and cervical insufficiency. Our patient suffered cervical insufficiency in the second trimester, which may be related to hysteroscopic dilatation. Therefore, the use of smaller diameter hysteroscopy can be considered in future treatment.

During the operation, the pressure and flow rate of dilation was maintained as low as possible to avoid too much dilation fluid entering the uterine cavity and adverse effects on intrauterine pregnancy. Ultrasound monitoring ensures that fluid flowing into the uterine cavity does not exceed one-third of the uterine cavity volume at all times. In addition, during the procedure, if ultrasound shows that the fluid in the uterine cavity is about to reach the edge of the implantation site, hysteroscopy should be immediately suspended, and the fluid should be drained by dilating the internal cervix os. Dilatation of the internal cervical os can be done with oval forceps under ultrasound monitoring. When the intrauterine effusion is discharged, the hysteroscope can be re-inserted to continue the removal of residual cervical pregnancy tissue until complete removal.

In summary, cervical pregnancy is a rare but dangerous ectopic pregnancy, especially when combined with intrauterine pregnancy. At present, our diagnosis and treatment have greatly improved, and the success rate of treatment is very high. According to our case, hysteroscopic surgery is a completely feasible cervical pregnancy treatment option with less postoperative impact on the mother and the intrauterine fetus. There are many treatment options available for compound pregnancies, so in choosing a treatment option, we should first consider the patient’s condition and their expectations of the outcome, as well as the level of the treating physician and the hospital’s resources, to achieve the best possible outcome and long-term prognosis.

## Acknowledgments

We appreciate the patient’s willingness to provide her case.

## Author contributions

**Conceptualization:** Shuman Sheng, Haomeng Zhang, Fei Wang.

**Data curation:** Xin Wang.

**Funding acquisition:** Fei Wang.

**Methodology:** Tao Li.

**Project administration:** Shuman Sheng, Haomeng Zhang, Zhengwu Pan, Tao Li, Min Shi.

**Writing—original draft:** Shuman Sheng.

**Writing—review and editing:** Shuman Sheng, Fei Wang.
